# P-571. Assessment of Failures of Long-Acting Cabotegravir and Rilpivirine in a Real-World Treatment Setting

**DOI:** 10.1093/ofid/ofae631.769

**Published:** 2025-01-29

**Authors:** Nicholas F Yared, Smitha Gudipati, Shannon Payne, Indira Brar

**Affiliations:** Henry Ford Health System, Detroit, MI; Henry Ford Health System, Detroit, MI; Henry Ford Health, Detroit, Michigan; Henry Ford Hospital, Detroit, Michigan

## Abstract

**Background:**

Cabotegravir (CAB) + rilpivirine (RPV) is the first complete long-acting all injectable (LAI) antiretroviral therapy (ART). While well-tolerated with low risk of virologic failure (VF), understanding reasons for CAB + RPV (CAR) discontinuation in a real-world setting was the objective of our study, so as to better define appropriate patients for LAI.

**Figure:**
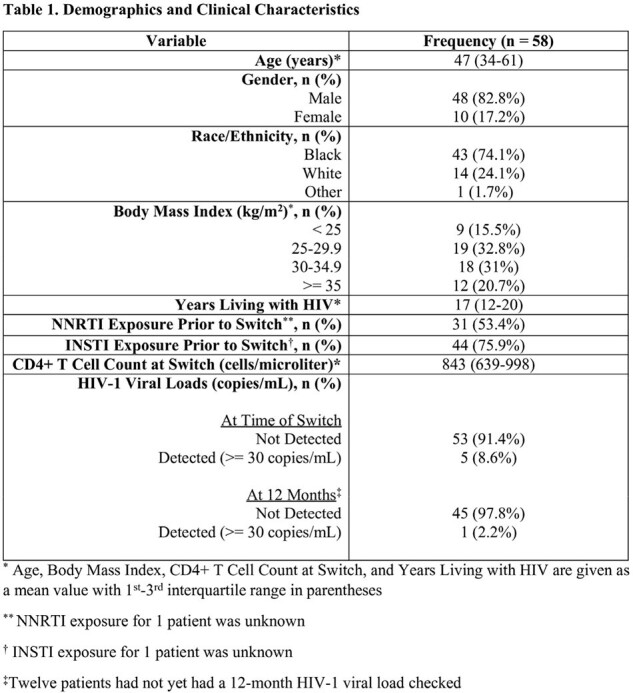

**Methods:**

We performed a retrospective cohort study to assess CAR failures. CAR failure was defined as regimen discontinuation after receipt of at least one CAR dose due to either virologic failure (VF) or side effects. VF was defined as viral load (VL) ≥ 30 copies/mL on 2 occasions at least 4 weeks apart. Genotypes incorporated detection of drug resistance mutations (DRMs) at frequency < 10%. We included PWH at Henry Ford Health who were virologically suppressed on their current ART and switched to CAR on or before March 2023. Demographics, clinical characteristics, and outcomes were extracted from the electronic medical record (Table 1).

**Figure:**
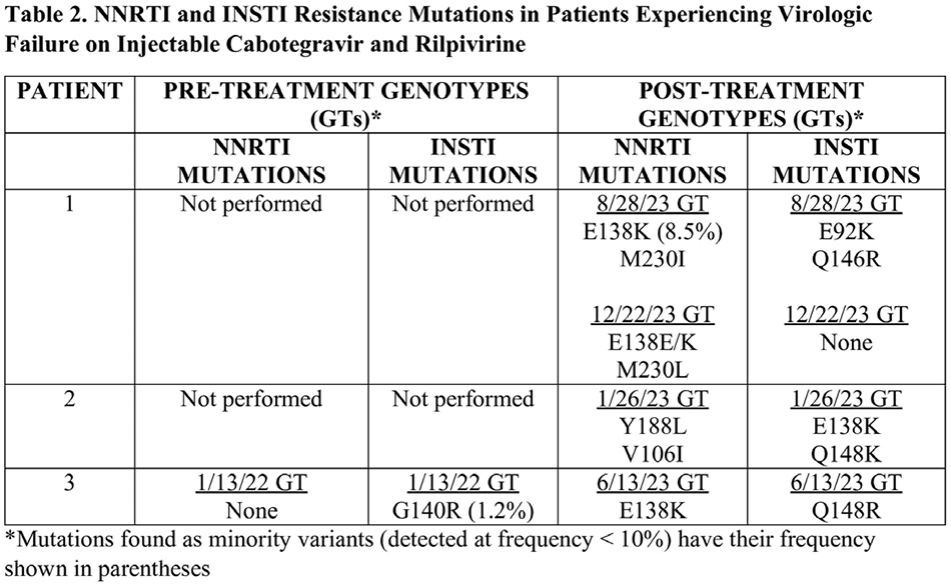

**Results:**

Of the 58 PWH initiating CAR, 46 had HIV VL testing available at 12 months after initiation of CAR and 45/46 PWH (97.8%) were undetectable (< 30 copies/mL). Five PWH failed CAR: 2/5 secondary to injection site reactions and 3/5 due to VF with new resistance (Table 2). Among those with VF, they had been living with HIV for a mean of 20 years and all had BMI > 30 kg/m^2^. One had a pre-existing G140R DRM (1.2% frequency) and developed the E138K NNRTI and Q148R INSTI DRMs ( > 10% frequency). A second patient developed the Y188L and E138E/K NNRTI and Q148K INSTI DRMs ( > 10% frequency). The third patient with history of oral NNRTI use developed the M230I and E138E/K NNRTI DRMs ( >10% frequency).

**Conclusion:**

In our clinic more patients (8.6%) discontinued CAR than had been reported in randomized clinical trials. While the clinical significance of < 10% DRMs is unclear, one patient with pre-existing G140R < 10% DRM developed VF despite on-time injections leading to loss of NNRTI and INSTI class efficacy. Previous studies have shown that the detection of drug-resistant < 10% DRMs in NNRTI significantly increases the risk of treatment failure. Larger studies are needed to determine the impact of HIV-1 < 10% DRMs for other classes of ARTs, such as INSTIs, as this may have a big impact while evaluating appropriateness of a patient for CAR.

**Disclosures:**

**Indira Brar, MD**, Gilead: Advisor/Consultant|Gilead: Grant/Research Support|Gilead: Honoraria|Merck: Grant/Research Support|ViiV Healthcare: Grant/Research Support|ViiV Healthcare: Honoraria

